# NUT carcinoma of the head and neck: A case report and literature review

**DOI:** 10.1002/pro6.1237

**Published:** 2024-09-01

**Authors:** Yue Zhao, Jun Zhang, Wenjun Liao, Jiayu Li, Shichuan Zhang

**Affiliations:** ^1^ Department of Radiation Oncology Radiation Oncology Key Laboratory of Sichuan Province Sichuan Clinical Research Center for Cancer Sichuan Cancer Hospital &Institute Sichuan Cancer Center Affiliated Cancer Hospital of University of Electronic Science and Technology of China Chengdu Sichuan China; ^2^ Department of Pathology Sichuan Clinical Research Center for Cancer Sichuan Cancer Hospital & Institute Sichuan Cancer Center Affiliated Cancer Hospital of University of Electronic Science and Technology of China Chengdu China

**Keywords:** chromosome 15q14, head and neck, nuclear protein in testis carcinoma, radiation therapy

## Abstract

Nuclear protein in testis (NUT) carcinoma is a rare and highly aggressive cancer, characterized by rearrangements involving the *NUT* gene located on chromosome 15q14. In this report, we present the case of a 52‐year‐old female diagnosed with primary parotid NUT carcinoma. Despite undergoing surgery, adjuvant chemotherapy, and incomplete regional radiotherapy, the patient succumbed to the disease after an overall survival duration of 7 months. We retrospectively discuss patient clinical and pathological features, as well as therapeutic approaches of NUT carcinoma of the head and neck.

## Graphical abstract

Therapies in NUT carcinoma.

A comprehensive treatment approach is recommended for NUT carcinoma including chemotherapy, radiotherapy, and occasionally surgical resection. Importantly, Bromodomain and Extraterminal motif (BET) inhibitors and histone deacetylase inhibitors (HDACi) are regarded as novel potential therapies

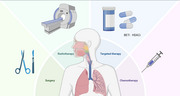

## 1 INTRODUCTION

Nuclear protein in testis (NUT) carcinoma is a rare and highly aggressive cancer defined by rearrangement of the *NUT* gene, which often arises from midline structures.^1^ NUT is a protein normally expressed only in the testis and ovary, encoded by *NUTM1* (NUT family member 1), which is located on the long arm of chromosome 15. This poorly differentiated neoplasm is characterized by the rearrangement of NUT involving other partner genes, resulting in a fusion protein that disrupts squamous cell differentiation and promotes the proliferation of immature neoplastic cells.^2^, ^3^ As one of the most aggressive forms of squamous carcinoma, more than 80% of patients with NUT carcinoma die within one year, with a median overall survival (mOS) of just 6.7 months.^4^

The World Health Organization (WHO) launched the 5th edition of Lung Cancer classification in 2015, identifying primary NUT carcinoma of lungs as a type of undifferentiated lung malignancy. In 2022, the WHO expanded its classification to include NUT carcinoma in the nasal cavity and paranasal sinuses.^5^, ^6^ NUT carcinoma used to be referred to as NUT midline carcinoma due to its prevalence in midline structures. However, NUT carcinoma is not confined only to midline organs. Cases of salivary gland, larynx, oral cavity, central nervous system (CNS), as well as ovary and kidney have also been reported.^7^ In this paper, we present a case of NUT carcinoma of the parotid gland. Additionally, we summarize the common molecular patterns, treatment strategies, and potential clinical trials for NUT carcinoma of the head and neck, with the aim of providing insights for future clinical trial design.

### 1.1 Case presentation

**FIGURE 1:**
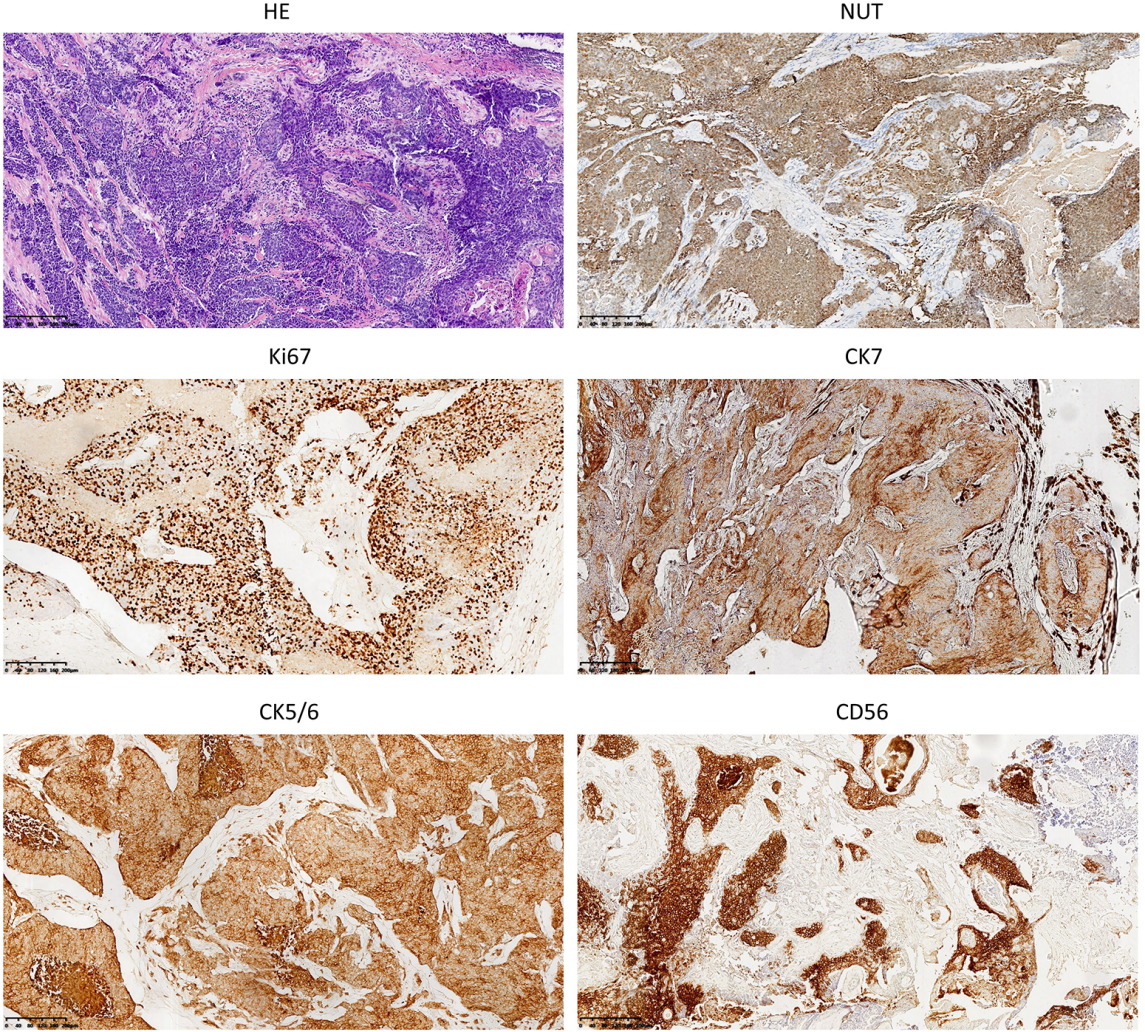
Pathological characteristcs of the case.

**FIGURE 2:**
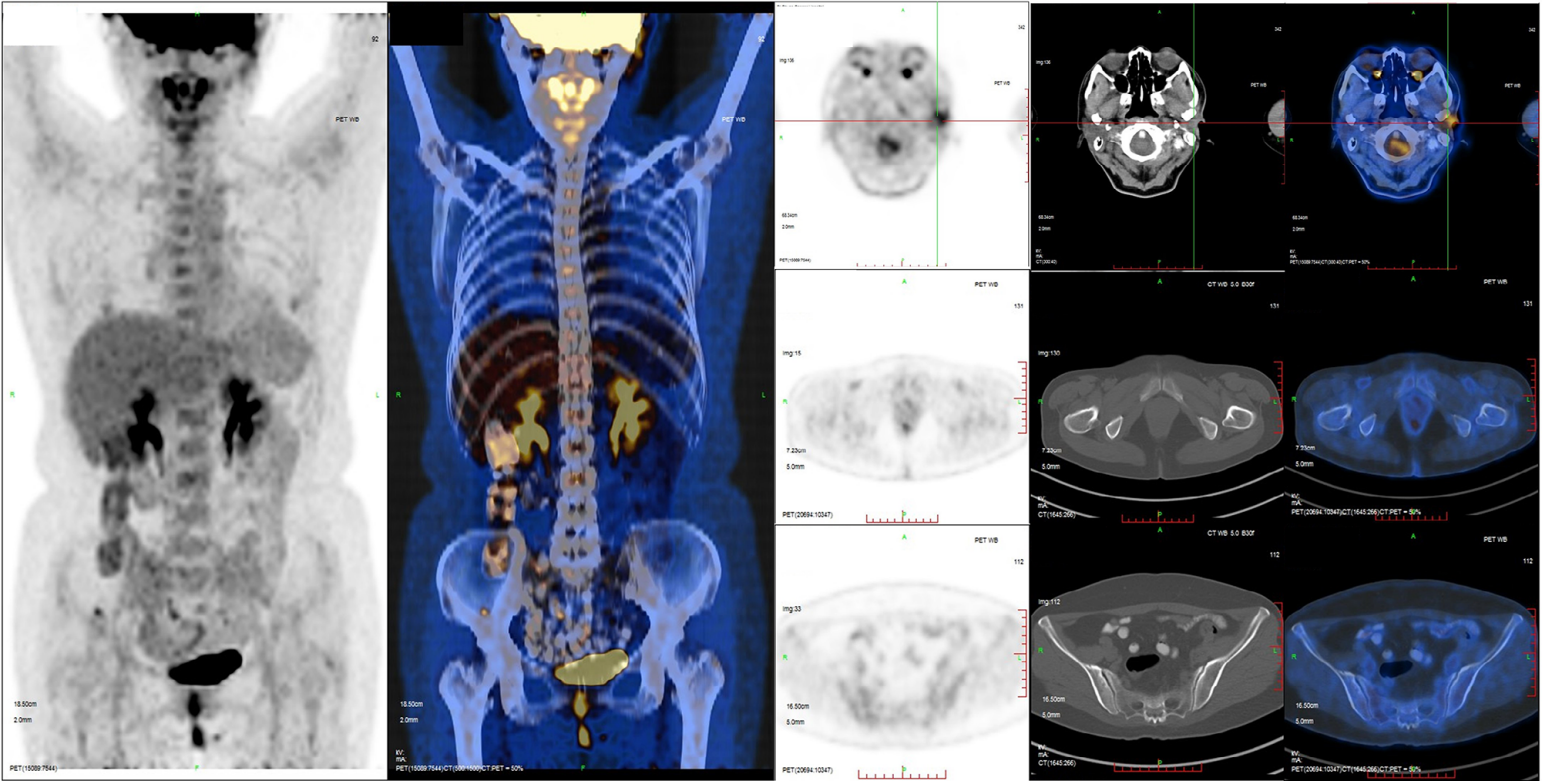
Post‐surgical PET/CT images of the patient.

In November 2020, a 52‐year‐old woman was admitted to hospital with the chief complaint of a nodule near her left ear that had been present for one month. She had no medical conditions or previous surgical history, and she had never smoked or abused alcohol. Ultrasonography revealed a low‐echo nodule (4.1 cm x 2.4 cm) with abundant vascularity in the left parotid gland. CT scans also confirmed an isolated infiltrative nodule in the left parotid gland. The mass was initially suspected to be a benign parotid gland tumor, with differential diagnoses including adenoid cystic carcinoma and mucoepidermoid carcinoma. The patient underwent a superficial lobe parotidectomy. Biopsy specimens of the tumor tissue revealed a cluster of undifferentiated small round cells that were positive for PCK, P63, C5/6, and CK7, but negative for CEA, TTF‐1, CGA, and Syn on immunohistochemistry (Figure 1). Importantly, the cells were positive for NUT expression. Further FISH analysis confirmed a *NUT* gene translocation. Post‐surgical PET/CT suggested residual tumor involvement in the mastoid process of the mandible and in the mastoid process of the temporal bone. No signs of metastasis were detected (Figure 2).

**FIGURE 3:**
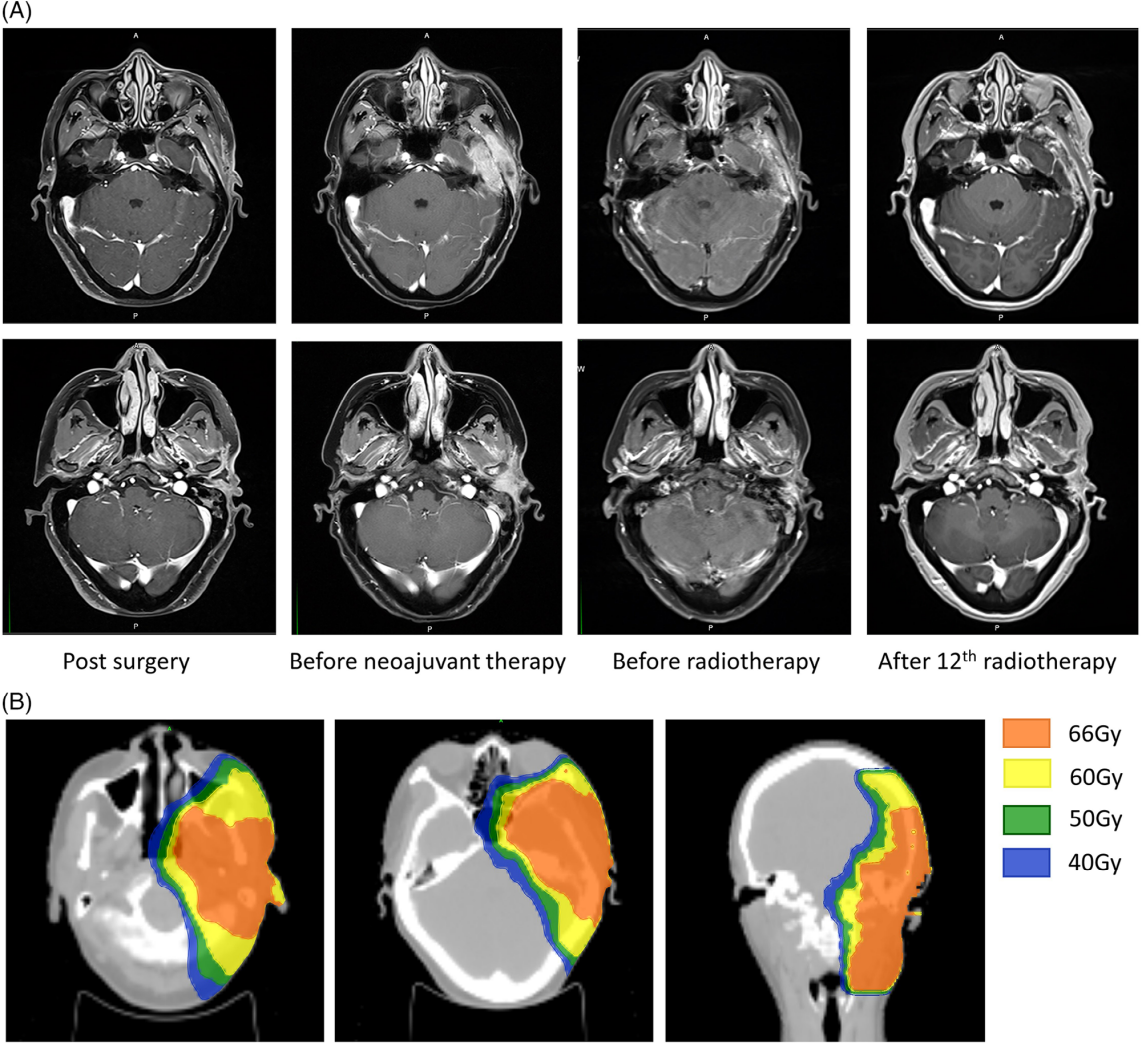
Representive magnetic resonance imaging during treatment (A) and isodose line of target volume images (B) of the patient.

In December 2020, a multidisciplinary team discussed treatment strategies, and the patient began first‐line therapy with paclitaxel and cisplatin (paclitaxel: 135 mg/m^2^; cisplatin: 75 mg/m^2^). However, subsequent follow‐up was delayed due to the COVID‐19 pandemic. When she returned in February 2021, MRI indicated rapid tumor progression involving the left temporal bone and neighboring meninges. Consequently, three additional cycles of cetuximab (400 mg/m^2^ first week, followed by 250 mg/m^2^ per week), nab‐paclitaxel, and cisplatin (nab‐paclitaxel: 260 mg/m^2^; cisplatin: 75 mg/m^2^, q3w) were administered. A follow‐up MRI of the head and neck indicated partial regression of the tumor (Figure 3A).

**FIGURE 4:**
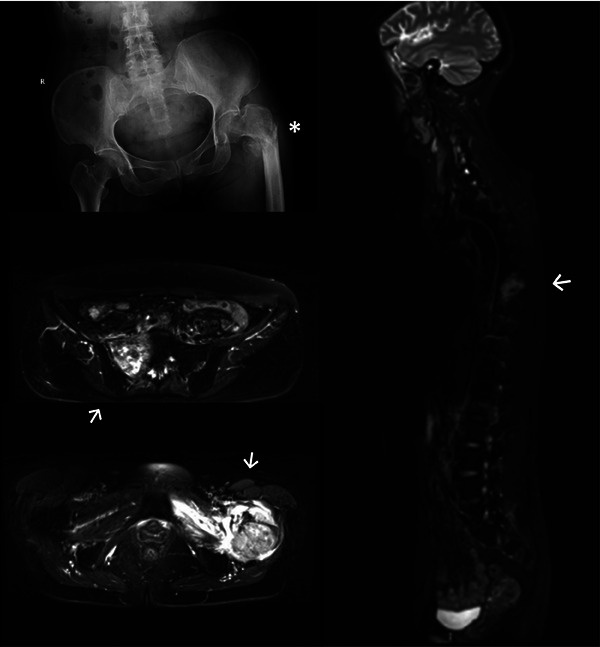
Digital radiography and magnetic resonance images of the bone fracture (*) and metastasis (→).

In April 2021, the patient was prescribed palliative radiotherapy for the left temporal bone and meninges, with a dosage of 66 Gy over 30 fractions (Figure 3B). Unfortunately, she experienced an unexpected pathological fracture of the left greater trochanter of the femur during her 12th radiation session (Figure 4). Consequently, the patient discontinued all anti‐cancer treatments and transitioned to hospice care. She passed away within two months due to tumor progression and multiple organ dysfunction syndrome (MODS).

## 2 DISCUSSION

### 2.1 Pathological characteristics and molecular patterns

NUT carcinoma typically consists of small‐to‐medium‐sized oval shaped cancer cells with irregular, often overlapping nuclei arranged in a nested pattern.^7^ This disease can be diagnosed using an anti‐NUT antibody, which provides 87% sensitivity and almost 100% specificity, with a positive predictive value of 100% and a negative predictive value of 99%.^4^, ^8^ Most cases (69−75%) express epithelial markers such as AE1‐AE3, CK7, CAM5.2, CK5‐6, CK20, and EMA, while other epithelial markers including P40 and P63 are discovered in approximately half of cases.^4^ Neuroendocrine markers (synaptophysin, NSE, chromogranin A, etc.) can be positive in 20% of cases. Additionally, focal expressions of CD99, TTF1, CD56, and CD138 can also be detected.^9^, ^10^

NUT carcinoma is characterized by translocations involving the *NUTM1* gene (located on chromosome 15q14) and its fusion with other partner genes. The most frequent partner gene is the chromatin‐binding bromodomain and extra‐terminal (BET) protein *BRD4* (chromosome 19), accounting for 75% of all cases. Other partner genes involved in encoding BRD4‐related proteins include *BRD3* (chromosome 9, 15%), *NSD3* (chromosome 8, 5%), *ZNF532* (chromosome 18), *ZNF592* (chromosome 15), and *CIC* (chromosome 19) (5% of all cases).^4^, ^11^, ^12^, ^13^, ^14^, ^15^ It is noteworthy that gene fusions involving *NUTM1* are not restricted to NUT carcinoma. For instance, *YAP1‐NUTM1* gene fusion has been reported in auditory canal carcinomas exhibiting features of porocarcinoma.^16^, ^17^ *PARD3B‐NUTM1* fusion has been detected in aggressive primary CNS embryonal tumors. Additionally, thyroid carcinoma has been associated with *NUT* rearrangements, showing thyrocyte differentiation while lacking squamous cell differentiation.^10^, ^18^ A rare case of high‐grade spindle cell sarcoma of the scalp with an *MGA‐NUTM1* gene fusion has also been reported.^19^ The less‐common fusion partner genes functionally interact with BRD4, highlighting the role of BET family protein‐mediated recruitment of NUT to chromatin in NUT carcinoma pathogenesis. Consequently, BET inhibitors (BETi) and HDAC inhibitors (HDACi) are two mainstream therapeutic strategies for treating NUT carcinoma.

### 2.2 Status quo of treatment strategies in NUT carcinoma of head and neck

Although NUT carcinoma may affect patients in all age groups, the disease is characterized by prevalence in the adolescent and young adult population.^20^ The incidence of NUT carcinoma of the head and neck region is approximately 41%, second only to the mediastinum and pulmonary area, which accounts for 51% of cases. NUT carcinoma of the head and neck primarily occurs in the nasal sinus region, larynx, and salivary glands. The most frequently metastatic site is bone. Other common metastasis sites include liver, adrenal gland, and lungs.^7^ Currently, there are no specific treatment options for NUT carcinoma patients. A comprehensive treatment approach, often including chemotherapy, radiotherapy, and occasionally surgical resection, is typically considered.

As suggested at the 2022 NUT Symposium, a therapeutic paradigm was proposed for head and neck NUT carcinoma.^20^ For localized, resectable disease, the recommendation is radical surgery followed by adjuvant chemoradiotherapy. For unresectable localized disease, definitive chemoradiation is suggested irrespective of whether upfront induction chemotherapy was conducted or not. However, chemotherapy with or without palliative radiotherapy is recommended for extensive disease.^20^ In a systemic review of 119 cases involving all primary sites, mOS for this group was only 5 months. Univariate analysis indicates that radiotherapy as well as chemotherapy as primary treatment strategies remarkably impact on overall survival (OS), yet surgery does not influence mOS significantly.^21^ However, for localized disease of head and neck, upfront radical surgical resection is associated with superior 2‐year progression‐free survival (PFS) (*p* = 0.01) and OS (*p* = 0.01). Hence, for resectable disease of the head and neck, this approach is recommended.^20^, ^22^

Considering the benefits of clinical outcomes (pain relief, organ function retention, and quality of life improvement), radical radiotherapy is particularly recommended for patients with limited‐stage NUT carcinoma involving the head and neck.^23^ A systematic review of sinonasal NUT carcinoma indicated that radiation was part of the initial therapy in 8 of 25 cases. Six patients received radiotherapy as a second‐line treatment.^24^ In one case of laryngeal NUT carcinoma, the patient survived for 26 months after definitive radiotherapy with a dose of 60 Gy.^23^ Another systematic review of NUT carcinoma revealed that among 40 cases with head and neck origin, only 9 patients underwent definitive radiation with radiation doses from 50 to 70 Gy (median dose: 64.5 Gy).^21^

In a postoperative setting, 9 patients, including three with positive margins, received radiation doses ranging from 24 to 66 Gy (median dose: 63 Gy).^21^

### 2.3 Potential therapy and ongoing clinical trials

BRD4‐NUT has great therapeutic potential in using the BETi class of selective epigenetic regulators.^20^ New molecularly targeted therapies, such as HDACi, have shown promising results and are gaining traction.^25^, ^26^, ^27^ Mechanistically BETi are acetyl‐histone mimetic drugs that competitively inhibit the binding of BET subunit proteins (BRD4 or BRD3), bromodomains including (BD1 and BD2) to chromatin, thereby preventing the activation of proto‐oncogenes.^28^, ^29^ NUT‐fusion proteins drive the formation of hyperacetylated chromatin “megadomains” or “superenhancers,” including the enhancer regions of genes such as *MYC* and *TP63*, which inevitably promote tumor growth and prevent differentiation.^29^, ^30^ Several clinical trials of BETi have been conducted in NUT carcinoma patients, including Birabresib (MK‐8628/OTX015), RO6870810, BI 894999 and Molibresib (GSK525762), although some trials were canceled due to the rarity of the disease or adverse effects.^31^ In the first reported safety profile of the BETi birabresib, reversible thrombocytopenia was observed as the major dose‐limiting toxicity (DLT). Durations of responses were also detected in NUT carcinoma patients, ranging from 1.4 to 8.4 months.^32^ The most common treatment‐related adverse events of Molibresib were thrombocytopenia (64%), nausea (43%) and decreased appetite (37%). Among 19 patients with NUT carcinomain a phase I study, 12 patients achieved objective responses, including four partial response (PR) cases, and eight had stable disease: among the latter, 4 patients were progression‐free for more than 6 months.^33^ In an expanded cohort of this study, 1/12 patients (8%) with NUT carcinoma achieved a confirmed PR with a median PFS of 4.8 months and the mOS was 5.0 months.^34^ The safety profile of BI 894999 was consistent with other BET inhibitors, including myelosuppression, hematological, gastrointestinal, and skin disorders, fatigue, and non‐hematological laboratory abnormalities, although the efficacy in NUT carcinoma was not disclosed.^35^ The most common AEs for another BETi RO6870810 includes fatigue, loss of appetite, and erythema at injection‐site. In NUT carcinoma cohort, the objective response rate (ORR) was 25%. Two patients achieved PR, while five patients had stable disease (SD), and one patient had PD as the best response, respectively. The median PFS in the NUT carcinoma group was 94 days (range, 15−783 days).^36^ The efficacy and response durability of BETi therapy may be further improved by multiple combination strategies. Currently, another trial to test for the efficacy of the BETi ZEN003694, either in combination with etoposide and cisplatin or abemaciclib, in individual patients is available (ClinicalTrials.gov Identifiers: NCT05019716 and NCT05372640) (Table 1).

**TABLE 1:** Clinical trials of nuclear protein in testis carcinoma.

NCT identifier	Drug	target	phase	status	Publication/Result
NCT01987362	RO6870810	BET Inhibitor	I	Completed	^36^
NCT04116359	GSK52576 +EP	BET Inhibitor	I/II	Withdrawn (Other—Protocol moved to Disapproved)	NA
NCT05019716	ZEN003694 +EP	BET Inhibitor	I/II	Recruiting	NA
NCT05372640	ZEN003694 and Abemaciclib	BET Inhibitor CDK4/6 Inhibitor	I	Recruiting	NA
NCT02516553	BI 894999	BET Inhibitor	Ia/Ib	Completed	^35^
NCT05488548	EP31670	BET Inhibitor CBP/p300 Inhibitor	I	Recruiting	^NA^
NCT03702036	GSK52576	BET Inhibitor	n/a	No longer available	^NA^
NCT01587703	GSK52576	BET Inhibitor	I/II	Completed	^33^, ^34^
NCT02307240	CUDC‐907	HDAC/PI3K Inhibitor	I	Completed	^NA^
NCT02698176	OTX015/MK‐8628	BET Inhibitor	I	Terminated (due to limited efficacy and not due to safety reasons)	^NA^
NCT02259114	OTX015/MK8628	BET Inhibitor	Ib	Completed	^32^
NCT02369029	BAY1238097	BET Inhibitor	I	Terminated	NA
NCT02711137	INCB057643	BET Inhibitor	I/II	Terminated	NA
NCT02431260	INCB054329	BET Inhibitor	I/II	Terminated	NA

Abbreviations: BET, bromodomain and extra‐terminal;CDK4/6, Cyclin‐dependent kinases 4 and 6; HDAC, Histone Deacetylase.

In addition to BETi, HDACi have also demonstrated efficacy in NUT carcinoma both in preclinical and clinical settings. HDACs play crucial roles in regulating cellular characteristics related to the development and progression of cancer.^37^ Enhanced HDAC activity leads to comprehensive genome hypoacetylation, is common in NUT carcinoma patients, and results in the transcriptional suppression of genes that regulate cell differentiation. Notably, a recent study on parotid NUT carcinoma associated with the overexpression of HDAC2, HDAC4, HDAC6, and phosphorylated HDAC4 (pHDAC457) documented the longest survival rates—with a disease‐free survival (DFS) of at least 47 months following diagnosis—reported in the medical literature.^29^ Furthermore, significant synergy between EZH2 inhibitors (EZH2i) and BETi was observed both in vitro and in vivo, promoting terminal squamous cell differentiation and arresting cell proliferation.^38^ Additionally, molecular signaling between BET proteins and HDACs underscores the potential for effective combined inhibition in malignancies, as already shown in vitro.^29^, ^39^

This finding holds promise for the potential of dual inhibition by BETi and HDACi—either alone or in combination with other regimens—in multiple clinical trials.

## 3 CONCLUSION

NUT carcinoma is a rare and aggressive malignancy that primarily affects midline organs, including the head and neck. This case report underscores the importance of regularly assessing tumor burden, even in instances where local disease control appears satisfactory. Enhanced understanding of the clinicopathologic characteristics of NUT carcinoma and its responsiveness to novel therapies could lead to improved clinical outcomes. However, the rarity of the disease may pose challenges to conducting randomized controlled trials.

## CONFLICT OF INTEREST STATEMENT

The authors declare that they have read the article and there are no competing interests.

## ETHICS STATEMENT

Written informed consent was obtained from a legally authorized representative for anonymized patient information in this case report to publish this paper. Approval to report this case was obtained from the Ethics Committee of Sichuan Cancer Hospital.
